# Leptin Modifies the Rat Heart Performance Associated with Mitochondrial Dysfunction Independently of Its Prohypertrophic Effects

**DOI:** 10.1155/2018/6081415

**Published:** 2018-08-01

**Authors:** Edna Berzabá-Evoli, Cecilia Zazueta, Jarumi Hishel Cruz Hernández, Nancy Patricia Gómez-Crisóstomo, Isela Esther Juárez-Rojop, Erick Natividad De la Cruz-Hernández, Eduardo Martínez-Abundis

**Affiliations:** ^1^Laboratory of Research in Metabolic and Infectious Diseases, Multidisciplinary Academic Division of Comalcalco, Juarez Autonomous University of Tabasco, Villahermosa, TAB, Mexico; ^2^Departamento de Biomedicina Cardiovascular, Instituto Nacional de Cardiología (I. Ch.), 14080 Tlalpan, MEX, Mexico; ^3^Research Center, Academic Division of Health Sciences (DACS), Juarez Autonomous University of Tabasco, Villahermosa, TAB, Mexico

## Abstract

**Background:**

Functional receptors for leptin were described on the surface of cardiomyocytes, and there was a prohypertrophic effect with high concentrations of the cytokine. Therefore, leptin could be a link between obesity and the prevalence of cardiovascular diseases. On the other hand, a deleterious effect of leptin on mitochondrial performance was described, which was also associated with the evolution of cardiac hypertrophy to heart failure. The goal of our study was to analyze the effect of the exposure of rat hearts to a high concentration of leptin on cardiac and mitochondrial function.

**Methods:**

Rat hearts were perfused continuously with or without 3.1 nM leptin for 1, 2, 3, or 4 hours. Homogenates and mitochondria were prepared by centrifugation and analyzed for cardiac actin, STAT3, and pSTAT3 by Western blotting, as well as for mitochondrial oxidative phosphorylation, membrane potential, swelling, calcium transport, and content of oxidized lipids.

**Results:**

In our results, leptin induced an increased rate-pressure product as a result of increased heart rate and contraction force, as well oxidative stress. In addition, mitochondrial dysfunction expressed as a loss of membrane potential, decreased ability for calcium transport and retention, faster swelling, and less respiratory control was observed.

**Conclusions:**

Our results support the role of leptin as a deleterious factor for cardiac function and indicates that mitochondrial dysfunction could be a trigger for cardiac hypertrophy and failure.

## 1. Introduction

Leptin is a 167-amino-acid-long peptide, with a molecular weight of 16 kDa. There is a direct correlation between the serum leptin concentration and body fat because leptin is mainly produced by adipocytes [1], reaching concentrations over 200 ng/mL in morbid-obese people compared with 10 ng/mL in thin people.

In addition to white fat, there are different tissues in the heart with the ability to synthesize and release leptin. The expression of leptin receptors on the cardiomyocyte surface has been described as well, suggesting a direct effect of the hormone on cardiac function [2, 3]. Results from previous studies indicate that high levels of serum leptin are positively associated with cardiac remodeling [4]. One of the first studies addressing the leptin effect on cardiac tissue found that 24 hours of treatment of neonatal isolated cardiomyocytes with high concentrations of leptin induced a 42% increase on the cellular surface [5], as well as the overexpression of alpha skeletal actin (*α*-actin), the myosin light chain 2 (MLC-2), and the atrial natriuretic peptide (ANP), all of which are considered to be hypertrophy markers. The average concentration leading to prohypertrophic effects was 3.1 nM (50 ng/mL). This group also demonstrated that the cardiomyocytes express the functional leptin receptors a and b (OB-Ra, OB-Rb) [5, 6]. Later studies from the same group found a mitochondrial deleterious effect of leptin that made cardiac mitochondria more sensitive to calcium overload [7]; additionally, there was evidence of OB-Rb in mitochondria isolated from rat hearts [8].

The heart is a high-energy-demand tissue; therefore, functional mitochondria are necessary for its correct function. In this context, any alteration in the mitochondrial performance induced by leptin will deeply affect heart functionality and may provide clues to explain the well-described relationship between obesity and cardiovascular diseases.

The goal of this study was to analyze the effects of the prohypertrophic concentration of leptin in an isolated heart model to demonstrate whether the *in vitro* deleterious effect also affects cardiac function by perfusion of hearts with leptin for 1, 2, 3, or 4 hours in an ex vivo model. We proposed that with heart being a high-energy-demand organ, a possible link between obesity and development of cardiovascular diseases might be related with direct effect of circulating leptin on mitochondria.

## 2. Materials and Methods

### 2.1. Ethics

Animals were purchased from the Production, Care and Animal Experimentation Unit (UPCEA), Juarez Autonomous University of Tabasco. Male Wistar rats weighing 300–350 g were used for this experimental design in compliance with the Mexican regulations for the use of animals in research (NORMA Oficial Mexicana NOM-062-ZOO-1999, technical specifications for production, use, and care of experimental animals), following the “Three Rs”: replacement, reduction, and refinement in research with animals.

### 2.2. Chemical Reagents

Primary and secondary antibodies were purchased from Santa Cruz Biotechnology (Dallas, Texas, USA). Luminol reagents for Western blotting were purchased from Thermo Scientific (Waltham, Massachusetts, USA). All remaining chemicals were purchased from Sigma-Aldrich (St. Louis, MO, USA) unless another source was described.

### 2.3. Heart Perfusion with Leptin

A Langendorff system for isolated hearts (Radnoti LTD, Ireland) was used for isolated-heart exposure to 3.1 nM leptin concentration as follows: rats were deeply anesthetized by intraperitoneal injection with sodium pentobarbital plus heparin (as an anticoagulant) before the heart was extracted by thoracotomy and quickly cannulated through the aorta in order to be continuously retrograde perfused with a physiologic Krebs-Henseleit buffer, pH 7.39, with or without leptin [9]. The buffer was oxygenated (O_2_95%/CO_2_5%), maintained at 37°C during the entire perfusion time (1, 2, 3, or 4 hours), and pumped at 12 mL/min. Cardiac performance was measured at left ventricular end-diastolic pressure (LVEDP) of 10 mmHg using a latex balloon inserted into the left ventricle and connected to a pressure transducer. The heart rate (beats per minute (bpm)) and contraction force (pressure (P)) have values which were used to calculate the rate pressure product.

### 2.4. Mitochondrial Isolation

After perfusion with or without leptin, the hearts were washed with cold STE isolation buffer (sucrose 250 mM, Tris-HCl 10 mM, and EDTA 1 mM), cut into small pieces, and incubated for 10 min with nagarse (1 mg/heart in cold STE). Nagarse was removed by centrifugation at 2500 ×g, and the tissue was homogenized in a Potter-Elvehjem homogenizer before a conventional method of centrifugation [10, 11], including 10 min incubation with 0.1% bovine serum albumin (BSA) in cold STE. Finally, the protein concentration was calculated with the Bradford method [12], using a standard curve with BSA as the standard.

### 2.5. Mitochondrial Oxygen Consumption

One milligram of isolated mitochondria was deposited on 1 mL of KHE buffer (KCl 130 mM, HEPES 25 mM, EGTA 0.1 mM, MgCl2 1 mM, and KH2PO4 3 mM) in a glass chamber adapted with a Clark-type electrode and constant stirring. State 4 respiration was evaluated [11] with either substrates for complex I (glutamate/malate) or complex II (succinate plus rotenone) using 2 mg of mitochondrial protein. State 3 of respiration was stimulated with the addition of 200 *μ*mol of ADP.

### 2.6. Mitochondrial Calcium Transport

A reduced ability to transport and accumulate calcium is a marker of mitochondrial dysfunction. We measured these mitochondrial features following the quenching of the calcium green fluorescence, as in previous reports [13, 14]. Five hundred milligrams of freshly isolated mitochondria was added to 1 mL of buffer C (KCl 130 mM, HEPES 25 mM, EGTA 0.1 mM, and KH2PO4 10 mM) supplemented with glutamate/malate as substrates for the mitochondrial complex I, 4 *μ*M calcium green, and 10 mM CaCl2. The reaction was stopped by the addition of a 0.1 mM aliquot of the mitochondrial uncoupler CCCP. Changes in the intensity of fluorescence were recorded by a spectrofluorometer with continuous stirring.

### 2.7. Mitochondrial Potential (ΔΨm)

The membrane potential was measured in the isolated mitochondria by monitoring changes in the fluorescence of the cationic dye Rhodamine 123, for which fluorescence is quenched as a result of mitochondrial accumulation of the dye [14, 15]. In brief, 0.5 mg of freshly isolated mitochondria was added to 2 mL of buffer C, before the addition of 0.1 mM rhodamine and 0.5 mM MgCl2. After a basal recording, the substrate glutamate/malate was added in order to induce potential formation, and finally, calcium and 10 mM or 0.1 mM CCCP were added where indicated.

### 2.8. Mitochondrial Swelling

Swelling was measured by conventional spectroscopy by monitoring the light absorption at 540 nm according to previous reports [16]. One milligram of isolated mitochondria was suspended in buffer C with glutamate/malate as a substrate, and swelling was induced by adding 10 mM CaCl2.

### 2.9. Western Blotting

Heart homogenates were mixed with loading buffer (glycerol 30%, SDS 10%, Tris 0.5 M, bromophenol blue 0.01%, and *β*-mercaptoethanol) and boiled for 10 min in a water bath. Proteins were separated by electrophoresis in 12% acrylamide/bis-acrylamide gels and transferred to nitrocellulose membranes [17]. After that, the membranes were blocked for 2 hours with 5% fat-free milk in washing buffer and incubated overnight at 4°C with the primary antibodies (mouse anticardiac actin, chicken anti-GAPDH, rabbit anti-STAT3, and mouse anti-p-STAT3) diluted 1 : 1000 with 3% BSA in washing solution. The next morning, the membranes were washed and incubated with the HRP-conjugated secondary antibodies diluted 1 : 10000 before detection with the Immobilon Western AP chemiluminescent substrate (Millipore, Bedford, MA, USA).

### 2.10. Determination of Oxidized Lipids

Lipid peroxidation was analyzed by the thiobarbituric acid-reactive substance (TBARS) methodology [18]. Isolated mitochondria or homogenate samples were mixed with TBA buffer and incubated for 20 min at 92°C, followed by 20 min of incubation in an ice bath, before centrifugation at 1200 ×g. The absorbance of the supernatants was measured at 532 nm using a thiobarbituric acid standard curve for the final calculations.

### 2.11. Statistical Analysis

Values were assessed by Student's *t* test and are given as the mean ± standard error (SE). A *p* value < 0.05 was considered to be the threshold for statistical significance between the compared groups. The correlation between variables was analyzed by Pearson's test. GraphPad Prism 6 was used for the statistical analysis, and ImageJ was used for the densitometry analysis of Western blots.

## 3. Results

### 3.1. Effect of Leptin on Cardiac Performance

**Figure 1:**
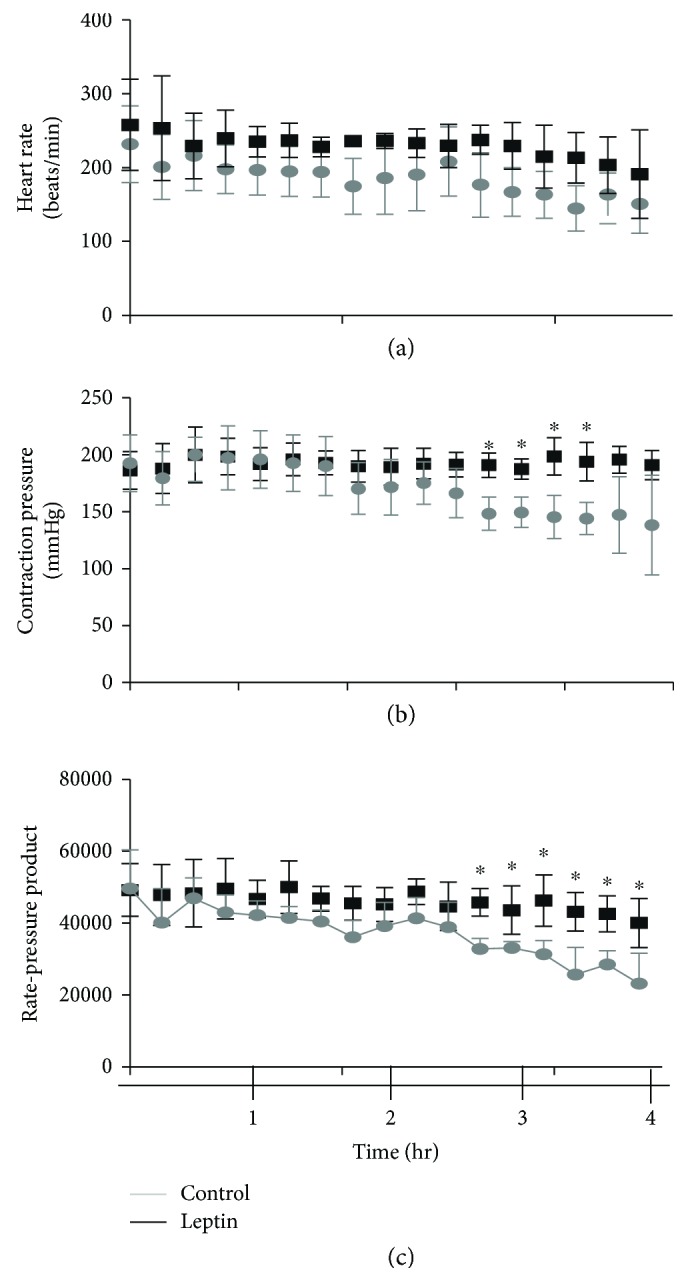
Effect of leptin on the cardiac rate-pressure product. (a) Plot of heart rates (beats per minute, mmHg) of leptin-perfused and control hearts. (b) Plot of heart contraction pressures of leptin-perfused and control hearts. (c) Heart rate-pressure product of leptin-perfused and control hearts; ^∗^*p* < 0.05 (*n* = 4). The concentration of leptin was 3.1 nM in the perfusion solution.

The first part of our study was focused on analysis of the effect of leptin on the rate-pressure product of the hearts. Rat hearts were perfused continuously with buffer containing or not 3.1 nM leptin. Figures 1(a) and 1(b) show an increasing mean value for the heart rate (HR) and the contraction pressure (CP) relative to the perfusion time, reaching statistical significance for CP after the third hour. Panel c in the same figure is the “rate-pressure product” (HR^∗^CP) and is indicative of the capacity of the heart for pumping. Note that leptin significantly increases this value after 3 hours of perfusion. These results are indicative of the direct and accumulative effect of these concentration of leptin on the heart function.

### 3.2. Effect of Leptin on Mitochondrial Function

The second set of the experiments was developed to analyze the effect of leptin on the mitochondrial function. Therefore, mitochondria were isolated from each control (Ctrl) or leptin-perfused heart (Lep) and analyzed by different approaches, yielding the following results.

#### 3.2.1. Respiratory Control

Oxidative phosphorylation is the main pathway for ATP synthesis, which, coupled with consumption of oxygen, is the foremost function of mitochondria. The uncoupling effect of leptin is evident from the first hour of perfusion.  Table 1 displays resumed data for respiratory control (RC), a value obtained by dividing the rate of oxygen consumption in state 3 by that of state 4. Leptin decreased the values of CR with substrates for either complex I (glutamate/malate, G + M) or complex II (succinate + rotenone (Succ)), indicating a lower efficiency for ATP synthesis and increased uncoupled respiration.

#### 3.2.2. Calcium Internalization and Retention

**Figure 2:**
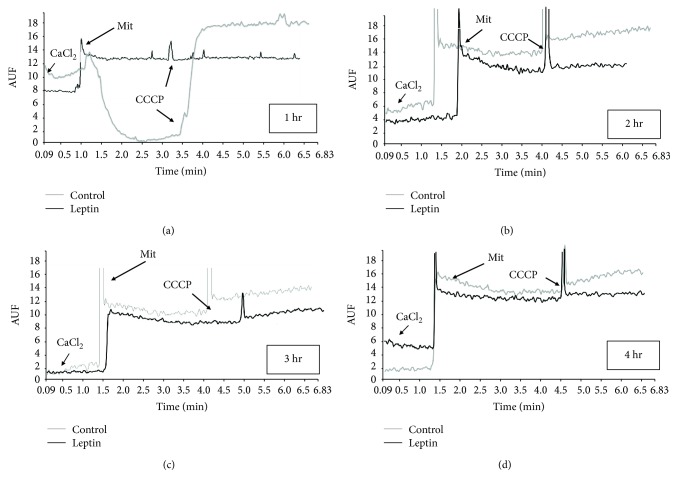
Calcium transported by mitochondria. (a) Plot of calcium internalization during active oxidative phosphorylation for control (gray) and leptin-treated (black) mitochondria. Arrows indicate the time points when the indicated additions were made. The reaction was stopped by the addition of CCCP to induce a complete release of calcium. (b), (c), and (d) are representative plots of control and leptin-treated mitochondria after 2, 3, and 4 hours of perfusion, respectively. CCCP: carbonyl cyanide-m-chlorophenyl hydrazone; Mit: mitochondria; AUF: arbitrary units of fluorescence.

The high capacity of mitochondria for calcium internalization and retention is highly important for other mitochondrial functions, for example, intracellular calcium buffering.  Figure 2 displays representative recordings of the fluorescence measurements as a result of mitochondrial calcium movements. In panel a, the ability of control mitochondria to internalize and retain calcium is clear; calcium is released just after membrane depolarization with the protonophore CCCP; however, mitochondria isolated from leptin-perfused hearts display a poor ability to transport and accumulate calcium, with no changes in the fluorescence. Panels b, c, and d show results from the same experiment after 2, 3, and 4 hours of perfusion, respectively. Unexpectedly, we detected a reduced capacity for calcium management in the control mitochondria, with little or null changes after CCCP addition; mitochondria from the leptin group showed the same profile with almost undetectable calcium retention.

#### 3.2.3. Membrane Potential (ΔΨm)

**Figure 3:**
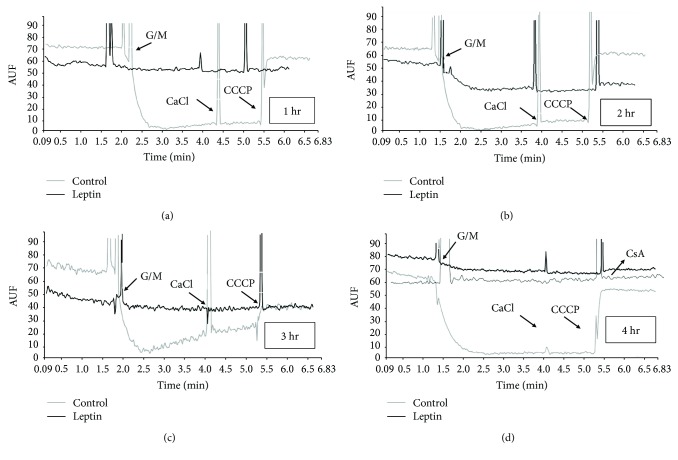
Determination of mitochondrial potential. (a) Representative plots of the experimental measurement of potential of control (gray) and leptin-treated (black) mitochondria during active oxidative phosphorylation. The reaction was started by the addition of glutamate/malate (G/M), and the potential was collapsed by the addition of CCCP. (b), (c), and (d) are representative plots of control and leptin-treated mitochondria after 2, 3, and 4 hours of perfusion, respectively. Panel d includes a plot of leptin-treated mitochondria treated with the mPTP inhibitor cyclosporine A (CsA). Arrows indicate the time points when the indicated additions were made. Note that the potential of leptin-treated mitochondria was neither established since the first hour of perfusion nor recovered with CsA.

**Figure 4:**
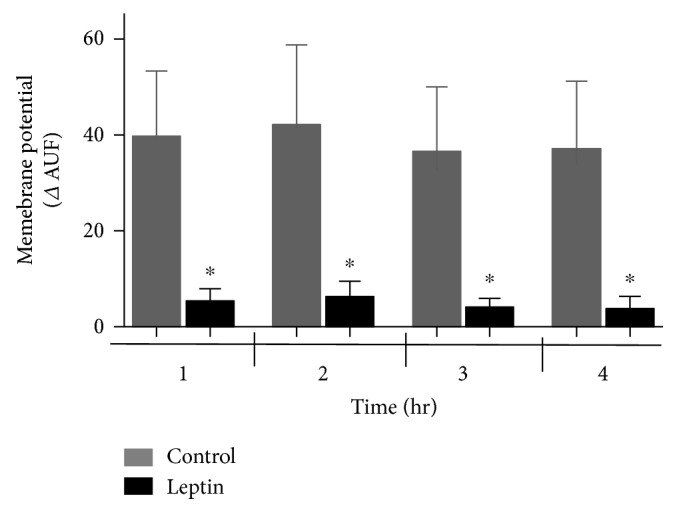
Semiquantitative analysis of the membrane potential of isolated mitochondria. Bars represent the median ± SEM of 4 independent determinations with different mitochondrial preparations. The delta AUF was measured after CCCP addition and was considered the membrane potential. ^∗^*p* < 0.05 (*n* = 4).

The mitochondrial membrane potential is indicative of the inner membrane integrity.  Figure 3 shows similar features between the membrane potential and calcium transport. In panel a, a large quenching of fluorescence was detected after the addition of mitochondrial substrates G + M to the control mitochondria, indicative of establishment of a potential, which was maintained upon addition of calcium but collapsed with CCCP, in contrast to the leptin-treated mitochondria that displayed a small decrease in fluorescence (smaller potential). The effect of leptin was maintained without changes even 2, 3, or 4 hours after perfusion (panels b, c, and d). Panel d includes a plot of leptin-treated mitochondria incubated with cyclosporine A (CsA), a potent inhibitor of mPTP. As noted, CsA did not correct the collapsed potential. For the semiquantitative comparison between groups, the potential was calculated as the difference of fluorescence before and after the addition of CCCP (in arbitrary units of fluorescence (AUF)) as displayed in Figure 4. As noted, the difference in the potential (measured as ΔAUF) was significantly higher after four hours of leptin perfusion.

#### 3.2.4. Mitochondrial Swelling

**Figure 5:**
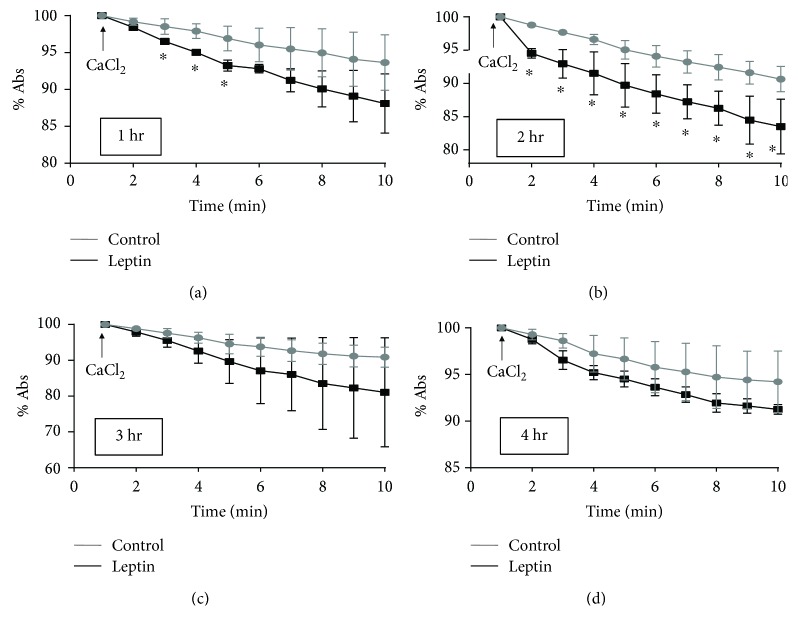
Mitochondrial swelling induced by calcium. Panels (a), (b), (c), and (d) are plots of the median ± SEM of 4 independent determinations of mitochondrial swelling of different mitochondrial preparations after 1, 2, 3, and 4 hours of perfusion, respectively. Reactions were started by the addition of CaCl2, and absorbance was recorded each minute for ten minutes. ^∗^*p* < 0.05 (*n* = 4).

The last experiments for the determination of mitochondrial performance investigated calcium-dependent swelling. Damaged mitochondria swell quickly when they are exposed to high concentrations of calcium. Figures 5(a)–5(d) show a faster decrease in absorbance in Lep-treated mitochondria than in control mitochondria as a result of calcium-induced swelling of the isolated mitochondria. Some of the plotted absorbance values are significantly different at the first and second hours, but such a difference disappeared at longer perfusion times (3rd and 4th hours). These results are similar to those of calcium transport, indicating a higher sensitivity of Lep-group mitochondria to calcium since the first hour of perfusion but also an effect on the control mitochondria at longer perfusion times.

### 3.3. Hypertrophy Determination

**Figure 6:**
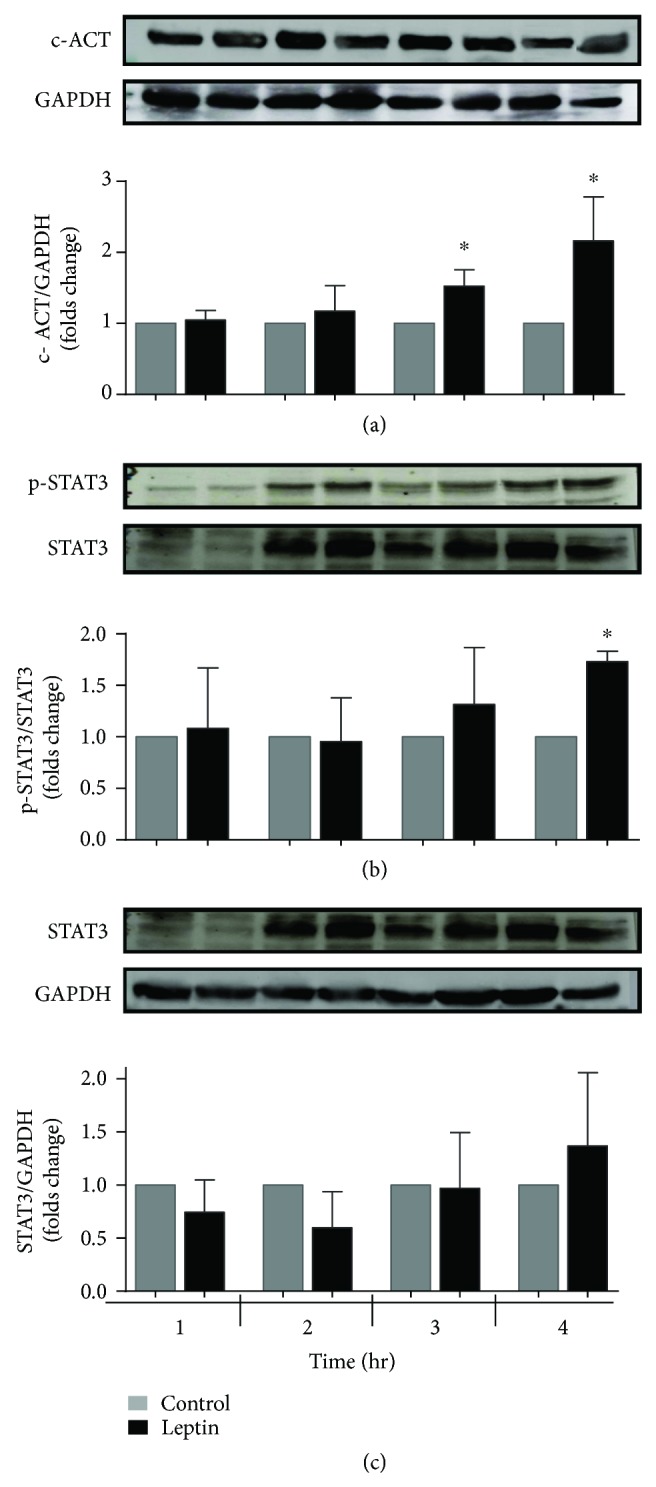
Leptin induces overexpression of the protein c-ACT and increases phosphorylation of STAT3. (a) The c-ACT protein content was measured by Western blotting of the heart homogenate using GAPDH as the loading control. Bars represent the median ± SEM of four independent mitochondrial preparations. (b) The phosphorylated STAT3 protein content was measured by Western blotting of the heart homogenate using total STAT3 as a reference control. (c) The STAT3 protein content was measured by Western blotting of the heart homogenate using GAPDH as the loading control. The values indicate the mean ± SEM, *n* = 4; ^∗^*p* < 0.05 versus the control group.

One of the main goals of our research was to determinate the prohypertrophic effect of leptin on the whole heart.  Figure 6(a) shows the results of a semiquantitative determination of cardiac actin (c-ACT), as a marker of heart hypertrophy. As noted, a significant increase in the amount of this protein was detected after 3 and 4 hours of perfusion in homogenates of these hearts. Such an increase was accompanied by phosphorylation of STAT3 (p-STAT3, Figure 6(b)), a signaling protein that is implicated in the prohypertrophic effect of leptin, but no changes were detected in the total protein (STAT3, panel c). These results suggest that 3 hours of perfusion with leptin are sufficient for the triggering of prohypertrophic signaling, in part through the STAT3 pathway.

### 3.4. Oxidative Stress

**Figure 7:**
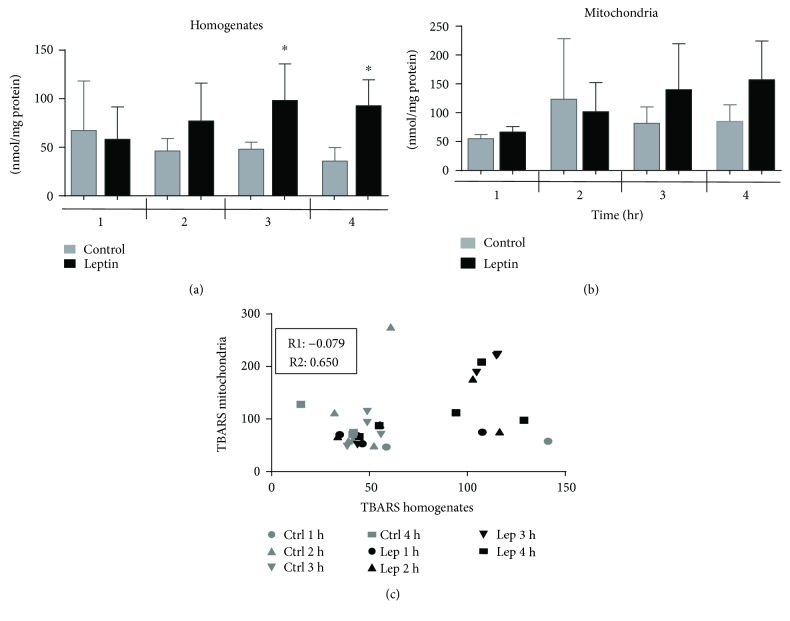
Quantification of oxidized lipids in heart homogenates and isolated mitochondria. Levels of oxidized lipids were measured by TBARS methodology in (a) homogenates after 1, 2, 4, or 4 hours of perfusion with/without leptin. Bars represent the median ± SEM of four independent preparations; ^∗^*p* < 0.05 vs the control group. (b) Isolated mitochondria. (c) Values of TBARS from homogenates and mitochondria were analyzed by Pearson's test to identify a correlation between variables. R1 = Pearson's value for control TBARS in the four time points analyzed; R2 = Pearson's value for leptin TBARS in the four time points analyzed. Note that the correlation is strong and positive for the leptin group.

One of the main consequences of mitochondrial damage is increased oxidative stress. We analyzed the level of oxidative stress in either the total tissue (homogenates) or isolated mitochondria with the TBARS technique, which quantifies the level of oxidized lipids. Our results plotted in Figure 7(a) show that the levels of these oxidized lipids in the heart homogenates are higher after the third and fourth hours of perfusion with leptin than in control groups. Interestingly, these results are similar to those obtained from the isolated mitochondria; however, in the organelle, the difference was not significantly higher. To determinate a cause-effect association, we calculated the correlation between the levels of oxidized lipids in the homogenates and in the mitochondria. As result, in panel C, the value of the correlation between the control samples is very low (*R* = −0.079) relative to that of the Lep group (*R* = 0.650), indicating that oxidative stress increases simultaneously in both mitochondria and whole tissue and explaining, at least in part, mitochondrial dysfunction.

## 4. Discussion

The goal of this study was to determine whether the exposure to a high concentration of leptin has a deleterious effect on the heart and mitochondrial performance using an isolated rat-heart model. Previously, the existence of leptin receptors in the heart was documented [15], suggesting a cardiac effect of this cytokine. These reports gained special interest in the context of obesity because obese people develop leptin resistance, with serum levels of leptin over 200 ng/mL. Although a cardioprotective effect of leptin was reported previously [19], the association between hyperleptinemia and development of cardiovascular diseases was described [20], particularly with congestive cardiac insufficiency and coronary disease [21, 22] in addition to the well-demonstrated prohypertrophic role on cardiomyocytes [5]. In our results, the hearts exposed to the prohypertrophic concentration of leptin developed an increase in either heart rate or ventricular pressure from the first to the fourth hours of exposure, suggesting an immediate effect, probably related to an increase in the intracellular concentration of calcium; this is similar to the constrictive effect that results from opening transient receptor potential cation channels (TRPCs) and voltage-dependent calcium channels and mobilizing intracellular calcium deposits, as described previously in thoracic aorta and lung artery from spontaneously hypertensive rats [23]. These results should be interpreted carefully since high values of rate-pressure product are not necessarily good for the heart. Interestingly, these effects occur simultaneously with mitochondrial dysfunction, measured as low respiratory control, decreased membrane potential, and reduced calcium transport (Figures 2 and 3). Our group reported previously that 24 hours of incubation with leptin sensitized mitochondria from isolated neonatal ventricular cardiomyocytes to calcium overload, mainly through opening of the mitochondrial permeability transition pore [8]. In this new approach, we were able to demonstrate that these deleterious effects occur in the whole heart earlier than that in isolated myocytes. This discrepancy is probably associated with the experimental model because in this report, hearts were beating continuously for the four hours, which suggests a higher mechanical wear that could favor mitochondrial damage.

There are several mechanisms for the establishment of mitochondrial dysfunction, that is, increased oxidative stress, which damages lipids and proteins, calcium overload, damage to mitochondrial respiratory chain complexes, or the loss of the mitochondrial inner membrane (MIM) integrity by swelling or pore formation. Our results showed an uncoupling effect of leptin on oxidative phosphorylation (respiratory control), which affected oxygen consumption through either complex I or II (Table 1), suggesting that the function of other complexes may be affected; additionally, the absence of oxidative stress between the first two hours, which has a greater effect on complex I, supports this hypothesis (Figure 7). On the other hand, either the ability of mitochondria to internalize and retain calcium or the establishment of a potential was affected (Figures 2–5), resulting, again, in the uncoupling of oxidative phosphorylation (oxygen consumption without ATP syntheses) and mitochondrial swelling. These last effects have been attributed to the opening of MIM pores/channels, such as mPTP or the mitochondrial apoptosis-induced channel (MAC) [24, 25], which could be activated by a calcium overload, oxidative stress, or apoptotic signaling [26]. In order to test this point, we added the mPTP inhibitor CsA to leptin-exposed mitochondria. Interestingly, the collapsed potential was not corrected (Figure 3(d)), which is a clear discrepancy with our study on the isolated myocytes, where sanglifehrin A, another mPTP inhibitor, completely prevented MIM permeation [8]. Another likely explanation is the activation of MAC, which is regulated by the proapoptotic members of the Bcl-2 family of proteins, Bax and Bak, and plays a key role in the intrinsic pathway of apoptosis [27, 28]. In addition, MAC could be activated and is more sensitive to both calcium overload and oxidative stress [28, 29]. Under specific circumstances, it is also possible that both pores are activated simultaneously, as we demonstrated previously [30]. It is important to point out that mitochondrial “dysfunction” does not mean that the mitochondrion is completely unable to synthetize ATP, but that means the functionality is affected or the organelle is sensitized to insults like calcium overload which is used in some of the experimental protocols; besides, the isolation process itself affects the organelle as can be appreciated with the control group. We did not develop a specific experiment analyzing integrity of the isolated mitochondria; however, in a previous report [7], it was demonstrated that a direct exposition to leptin induces mitochondrial swelling for itself and these mitochondria are more sensitive to calcium overload. On the other hand, although the detailed mechanisms to explain the effect of leptin on the electric properties of the heart are not well described. Lin et al. described a stimulant effect on the adrenergic receptors because a high concentration of leptin increases the QT interval, triggering arrhythmias such as premature ventricular bits, sinus pauses, and ventricular tachycardia [31]. Our results indicate that leptin induces alterations in cardiac performance, probably through upregulation of different calcium channels such as the L-type or Na/Ca translocator, resulting in calcium overload in mitochondria during contractile function and explaining the *in vitro* sensitization to calcium, but this remains a topic for future research.

## 5. Conclusions

In conclusion, our results strongly support previous reports that noted the deleterious effect of a high concentration of leptin on cardiac mitochondrial function but with a different mechanistic pathway (without participation of the mPTP) than that observed for isolated myocytes. Furthermore, mitochondrial dysfunction is an earlier event than the prohypertrophic effect, associated with the alteration of the electrical function, reinforcing the hypothesis that leptin-induced mitochondrial dysfunction contributes to hypertrophy and heart failure as a triggering and early phenomenon during obesity.

## 6. Limitations of the Study

The demonstration of the role of MAC is missing in our report. The swelling and collapse of the potential of both leptin and control mitochondria after hours of perfusion suggest that the experimental methodology had damaging affects in addition to the effects of the treatment.

## Figures and Tables

**Table 1:** Summary of respiratory control (RC) measured by conventional oximetry with a Clark-type electrode in isolated mitochondria. Oxygen consumption was analyzed with substrates for complex I (glutamate/malate) and complex II (succinate + rotenone) by measuring the slopes of state 4 and state 3, as shown in Figure 2. Values represent median ± SD; ^∗^*p* < 0.05 (*n* = 4).

Perfusion time (hr)	1		2		3		4	
	Ctrl	Lep	Ctrl	Lep	Ctrl	Lep	Ctrl	Lep
Glutamate-malate: (complex I)	2.07 ± 0.3	1.18 ± 0.1^∗^	2.26 ± 0.8	1.27 ± 0.3^∗^	1.80 ± 0.4	1.34 ± 0.1^∗^	2.96 ± 0.9	1.14 ± 0.1^∗^
Succinate: (complex II)	2.94 ± 1.1	1.28 ± 0.2^∗^	2.67 ± 0.8	1.19 ± 0.2^∗^	2.95 ± 0.5	1.15 ± 0.08^∗^	2.17 ± 0.7	1.18 ± 0.1^∗^

## Data Availability

The data used to support the findings of this study are available from the corresponding author upon request.
